# Prévalence et facteurs associés aux hépatites B et C chez les travailleuses du sexe de Bamako, Mali

**DOI:** 10.11604/pamj.2024.49.118.39119

**Published:** 2024-12-12

**Authors:** Gisèle Mukeya Mvumbi, Bintou Dembélé Keïta, Nana Camara, Ismaïla Théra, Fernand Aimé Guédou, Sory Traoré, Adam Yattasaye, Michel Alary, Souleymane Diabaté

**Affiliations:** 1 Centre de Recherche du CHU de Québec, Université Laval, Québec, Canada,; 2 Département de Médecine Sociale et Préventive, Faculté de Médecine, Université Laval, Québec, Canada,; 3 Association de Recherche, Communication et Accompagnement à Domicile des Personnes Vivant avec le VIH (ARCAD-SIDA), Bamako, Mali,; 4 Centre de Recherche et de Formation sur le Paludisme, Bamako, Mali,; 5 Dispensaire IST, Centre Communal de Santé de Cotonou-Zone 1, Cotonou, Bénin,; 6 Organisation pour la Promotion de la Santé et le Développement Communautaire, Cotonou, Bénin,; 7 Cellule Sectorielle de Lutte contre le VIH/SIDA, la Tuberculose et les Hépatites Virales, Ministère de la Santé, Bamako, Mali,; 8 Institut National de Santé Publique du Québec, Québec, Canada

**Keywords:** Hépatite B, hépatite C, VIH, infections sexuellement transmissibles, travailleuses du sexe, Bamako, Mali, Hepatitis B, hepatitis C, HIV, sexually transmitted infections, female sex workers, Bamako, Mali

## Abstract

**Introduction:**

les hépatites B (VHB) et C (VHC) constituent un problème de santé publique, particulièrement dans les pays à faible ou moyen revenu. Au Mali, Afrique de l'Ouest, peu de données existent sur la prévalence de ces hépatites en fonction du statut sérologique du virus de l'Immunodéficience Humaine (VIH) chez les groupes vulnérables comme les travailleuses du sexe (TS). Cette étude transversale conduite de mars à octobre 2020 à Bamako, au Mali, chez 400 TS (200 VIH+ et 200 VIH-) avait pour but de déterminer la prévalence et les facteurs associés à l'antigène HBs et aux anticorps anti-VHC.

**Méthodes:**

un questionnaire a été administré, des prélèvements sanguins et un auto-prélèvement vaginal ont été effectués. Les prévalences ont été présentées selon le statut du VIH. La régression logistique multivariée a été utilisée pour identifier les déterminants du VHB et du VHC.

**Résultats:**

la prévalence de l'antigène HBs et des anticorps anti-VHC étaient, respectivement, de 6,6% et 8,6 % chez les VIH+ et 4,6% et 6,1% chez les VIH-. En analyses multivariées, l'âge au premier rapport sexuel payant (<18 ans) et la présence d'anticorps anti-VHC étaient fortement associés au VHB (Rapport de cotes ajusté [RCa]; intervalle de confiance à 95% [IC95%]: 3,3; 1,24-8,63 et 3,7; 1,21-11,54, respectivement). Seul, l'antigène HBs était associé aux anticorps anti-VHC: RCa; IC 95%: 4,1; 1,29-12,56.

**Conclusion:**

afin de réduire le fardeau des hépatites B et C chez les TS de Bamako, un programme de prévention adapté à leurs conditions de vie et de travail est nécessaire.

## Abstract (en)

**Introduction:**

hepatitis B (HBV) and C (HCV) are a public health problem, particularly in low- and middle-income countries. In Mali, West Africa, few data exist on the prevalence of these infections among vulnerable groups such as female sex workers (FSWs) living or not with the human immunodeficiency virus (HIV). This cross-sectional study conducted from March to October 2020 in Bamako, main city of Mali, among 400 FSWs (200 HIV+ and 200 HIV-) aimed to determine the prevalence and factors associated with HBs antigen on one hand and HCV antibodies on the other.

**Methods:**

a questionnaire was administered, and blood and vaginal samples were collected. Prevalences are presented according to HIV status and multivariate logistic regression was used to assess the determinants of HBV and HCV.

**Results:**

the prevalence of HBs antigen and HCV antibodies were 6.6% and 8.6% in HIV+ and 4.6% and 6.1% in HIV- women, respectively. In multivariate analyses, age at first paid sexual intercourse (< 18 years) and presence of HCV antibodies were strongly associated with HBV (adjusted odds ratio [aOR]; 95% Confidence interval [95%CI]: 3.3; 1.24-8.63 and 3.7; 1.21-11.54, respectively). Only HBs antigen was associated with HCV antibodies (aOR; 95%CI: 4.1; 1.29-12.56).

**Conclusion:**

in Mali, the relatively high frequency of viral hepatitis B and C among FSWs requires a targeted prevention program.

## Introduction

Les hépatites virales B (VHB) et C (VHC) constituent un problème de santé publique international [1]. La distribution géographique de ces hépatites varie selon les régions et les sous-populations [1,2]. Selon les estimations de l'Organisation mondiale de la Santé (OMS) de 2020, le Mali était classé au 10^e^ rang mondial pour la mortalité liée au VHB avec un taux de décès ajusté pour l'âge de 1,97/100000 personnes-années [3]. Très peu de données existent sur la fréquence de ces hépatites chez les groupes les plus exposés au Mali et susceptibles d'entretenir la transmission au sein de la population générale. Ces groupes comprennent les donneurs réguliers de sang, les femmes enceintes, les hommes ayant des rapports sexuels avec d'autres hommes (HARSAH), les travailleuses du sexe (TS) et les utilisateurs de drogues injectables (UDI) [4]. La prévalence de ces hépatites dans la population des donneurs de sang du Mali est estimée à 10,7% pour le VHB et 1,5% pour le VHC [5]. Dans une étude chez des femmes enceintes en milieu rural au Mali, 8,0% de participantes étaient porteuses chroniques du VHB [6]. Une étude de démonstration sur l'utilisation de la prophylaxie pré-exposition (PrEP) pour la prévention du virus de l'immunodéficience humaine (VIH) par les HARSAH a été conduite entre 2017 et 2020 dans plusieurs pays de l'Afrique de l'Ouest (Etude Coh-MSM). Dans cette étude, la prévalence du VHB à Bamako-Mali était de 11,8% et celle du VHC de 0,7% [7]. Plusieurs facteurs peuvent expliquer que les TS soient plus susceptibles d'acquérir les hépatites B et C et de les transmettre à leurs clients et/ou partenaires réguliers [8]. Au Mali, les TS n'ont souvent pas accès aux services de dépistage communément organisés à l'intention des femmes de la population générale. Outre l'excision qui est une pratique très courante au Mali (89% des femmes de 15 à 49 ans) [9], d'autres procédures médico-chirurgicales ou esthétiques (interventions chirurgicales, curetage, tatouages, piercing) reconnues comme favorisant les infections par le VHB et VHC sont assez fréquentes chez les TS [9,10]. La morbidité élevée associée aux hépatites a conduit les autorités sanitaires et les autres parties prenantes maliennes à mettre en place des interventions de prévention basées sur des données probantes [8,10-12] et à inscrire ces hépatites virales comme une priorité de recherche dans le cadre d'un programme de recherche interventionnelle sur la santé sexuelle et l'équité pour les populations clés en Afrique de l'Ouest (POCAO) [12].

La présente étude qui a été conduite dans le cadre de ce programme avait pour objectif d'estimer la prévalence et d'analyser les facteurs associés à l'infection par le VHB et le VHC chez les TS de Bamako. Plus spécifiquement, il s'agissait de décrire les participantes de l'étude, d'estimer la prévalence de l'antigène (Ag) HBs et des anticorps (Ac) anti-VHC selon le statut sérologique du VIH, et d'identifier les facteurs associés à l'Ag HBs et aux Ac Anti-VHC. Nous avons émis l'hypothèse que la prévalence de l'Ag HBs et des Ac anti-VHC serait plus élevée chez les personnes dont le statut sérologique du VIH est positif.

## Méthodes

**Cadre de l'étude:** le cadre physique de l'étude comprenait quatre composantes: Le cadre communautaire composé des sites et points chauds fréquentés par les femmes TS et où étaient recrutées essentiellement les participantes VIH négatives; La clinique des Halles de l'Organisation non gouvernementale (ONG) ARCAD-SIDA où étaient recrutées essentiellement les participantes VIH positives et où se faisait la plupart des analyses de laboratoire (sérologie des hépatites B, C et du VIH); le Centre d'écoute, de soins et d'accompagnement des personnes vivant avec le VIH/SIDA (CESAC) où étaient recrutées des participantes VIH positives, en complément de celles recrutées à la Clinique des Halles pour atteindre le nombre requis de femmes; et le laboratoire AGIL où s'effectuait la *Polymerase Chain Reaction* pour le diagnostic de la gonococcie et de la chlamydiase.

**Population d'étude:** la population cible était constituée des TS de la ville de Bamako tandis que la population source regroupait les TS relevant des sites opérationnels des pairs éducateurs de ARCAD-SIDA, ainsi que les TS fréquentant les cliniques des Halles et du CESAC.

**Type d'étude:** il s'agissait d'une étude transversale, descriptive et à visée exploratoire conduite de mars à octobre 2020 à Bamako, Mali.

**Échantillonnage:** de manière générale, les participantes VIH négatives ont été recrutées sur les sites opérationnels de ARCAD-SIDA (recrutement de type communautaire) tandis que les VIH positives ont été recrutées dans les cliniques des Halles et du CESAC où elles étaient déjà connues et régulièrement suivies.

Pour être incluses dans l'étude, les participantes devaient remplir quatre critères: être considérée comme une TS c'est-à-dire une femme déclarant les rapports sexuels en échange d'argent ou de biens comme la principale source de revenus, soit plus de la moitié du revenu mensuel; résider à Bamako pendant au moins trois mois au moment du recrutement; opérer sur les sites couverts par ARCAD-SIDA et le CESAC; et être âgée d'au moins 18 ans. Toutes les TS qui ne pouvaient pas donner un consentement éclairé n'ont pas été retenues dans l'étude.

La taille de l'échantillon a été fixée à 400 participantes dont 200 dans le groupe VIH- et 200 dans le groupe VIH+. Avec un risque d'erreur alpha à 5%, une précision de 0,045 et 0,055, cet échantillon était suffisant pour détecter assez aisément (puissance statistique ≥ 0,80) des rapports de prévalences supérieurs ou égaux à 2,0 ou inférieurs ou égaux à 0,3, pour des facteurs dont la fréquence atteint au moins 30% [13].

**Collecte des données:** après l'obtention du consentement éclairé de la participante, une enquêtrice procédait à un entretien face-à-face à l'aide d'un questionnaire structuré. Au cours de celui-ci, elle collectait plusieurs données, y compris les caractéristiques comme l'âge, le pays d'origine, le niveau d'études, le statut matrimonial, le nombre d'enfants biologiques, le montant en Francs CFA réclamé par acte sexuel, l'ancienneté dans le travail du sexe et le principal lieu de sollicitation des clients. Après l'entretien avec l'enquêtrice, la participante était adressée à l'agent de santé qui collectait sur une « fiche biomédicale » des informations sur les antécédents médico-chirurgicaux (hémophilie, transfusion sanguine, interventions chirurgicales) et gynéco-obstétricaux (curetage utérin, césarienne). Un écouvillon était, par la suite, remis à la participante pour faire un auto-prélèvement vaginal pour la recherche des infections à *Neisseria gonorrhoeae* (*NG*) et *Chlamydia trachomatis* (*CT*) à l'aide du test CT/NG en temps réel d'Abbott (Abbott, Illinois, USA). Enfin, un échantillon sanguin veineux a été prélevé dans un tube lavande par un technicien de laboratoire à la recherche des marqueurs sérologiques des hépatites B et C (OnSite HBV-5 Rapid Test; CTK Biotech, San Diego, USA) et de l'infection par le VIH (test rapide Determine HIV-1/2 suivi de SD Bioline; Abbott, Illinois, USA).

**Analyse des données:** les données ont été enregistrées sur un fichier Excel et validées avant leur analyse statistique à l'aide du logiciel SAS, version 9.4 (SAS Institute Inc, Cary, NC, USA). Pour décrire les caractéristiques des participantes, les variables continues ont été présentées par leur médiane (intervalle interquartile, IIQ) et les variables catégorielles par leurs proportions. Pour les analyses comparatives de celles-ci, y compris les prévalences de l'Ag HBs et des Ac Anti-VHC selon le statut du VIH, un test du Khi-carré a été utilisé. Après cette étape, une analyse bivariée a été faite pour identifier les variables associées à l'Ag HBs et aux Ac anti-VHC. Le choix des variables indépendantes qui ont été considérées pour les analyses était basé sur la revue de la littérature. Tous les facteurs associés à chacune des deux variables dépendantes au seuil de signification de 20% ont ensuite été simultanément incluses dans des modèles multivariés. La méthode de sélection manuelle pas-à-pas a enfin servi à identifier le modèle multivarié ajusté final [13]. La régression logistique a été utilisée à la suite de la non-convergence du modèle de régression log-binomiale. Chaque rapport de cotes ajusté (RCa) a été présenté avec un intervalle de confiance à 95% (IC95%) et une valeur p (p).

**Considération éthique:** l'étude a été approuvée par le comité d'éthique pour la recherche en santé de la Faculté de Médecine et de Stomato-Odontologie de Bamako (No 2020-02-CE-FMOS-FAPH) ainsi que par le comité d'éthique du CHU de Québec-Université Laval (Projet 2020-5059/Approbation finale du 24 février 2020). Des explications claires sur les objectifs, les procédures et les risques d'ordre psycho-social chez les participantes testées séropositives ou d'ordre clinique avec la douleur au point de piqûre ont été fournies aux participantes. Ces dernières étaient libres de quitter l'étude à tout moment. Toute l'équipe de l'étude a été formée sur les principes fondamentaux de l'éthique de la recherche en santé. Les examens cliniques et les prélèvements ont été faits dans un cadre très confidentiel. Un formulaire de consentement signé a été obtenu avant l'inclusion dans l'étude.

Le coût élevé de la prise en charge médicamenteuse à vie de l'hépatites B et du traitement curatif de l'hépatite C (1,5 à 3 millions de Francs CFA par cas traité) ne permettaient pas à un projet de recherche de 11 mois d'offrir une prise en charge thérapeutique pour ces deux infections. En revanche, ces personnes ont été orientées vers les services appropriés pour leur prise en charge. De plus, le projet a pris en charge la vaccination des participantes qui étaient éligibles pour le vaccin contre l'hépatite B. Une compensation financière de 3000 Francs CFA a été remise à chacune des participantes pour couvrir les pertes d'opportunités de travail (recherche de clientèle) liées à la participation à l'étude. Les données ont été conservées pour une durée de 10 ans sur les serveurs sécurisés de banques de données à Bamako.

## Résultats

**Caractéristiques des participantes:** l'âge médian (IIQ) des femmes VIH+ et VIH- était de 30 ans (24-38) et de 22 ans (19-28), respectivement (Tableau 1). L'âge médian au premier rapport sexuel était de 16 ans (14-18) autant pour les TS VIH+ que pour les VIH-. Une femme sur cinq était originaire d'un pays autre que le Mali. Les femmes non scolarisées étaient plus nombreuses chez les VIH+ (41%) par rapport aux VIH- (29,5%). La moitié des TS séropositives au VIH et environ 4/5e des séronégatives étaient célibataires. La moitié des TS séropositives au VIH et seulement 23% des séronégatives avaient plus d'un enfant. Près de la moitié des TS (45,5% chez les VIH+ et 57,5 % chez les séronégatives au VIH) avaient comme source de revenu le travail du sexe uniquement. Le revenu mensuel médian (IIQ) était de 150 000 (100 000 - 250 000) FCFA dans les deux sous-groupes VIH+ et VIH-. La durée médiane en mois dans le travail du sexe était plus élevée chez les séropositives au VIH (48; IIQ, 24-84) par rapport aux séronégatives au VIH (36; IIQ, 18-60). La majorité des VIH+ (72,5%) et des VIH- (89,5%) a cité le bar comme principal lieu de sollicitation des clients. Le nombre médian (IIQ) de partenaires sexuels payants au cours des sept derniers jours était de 5 (3-10) chez les VIH+ contre 11 (4-14) chez les VIH- (Tableau 2). Deux femmes sur cinq ont rapporté n'avoir pas eu de nouveau client le dernier jour de travail. L'utilisation du préservatif avec le dernier client a été déclarée par la quasi-totalité des TS, indépendamment du sous-groupe de VIH. Si on considère les 400 femmes, moins d'un tiers avaient une scarification, plus d'un tiers avaient un tatouage et moins de 10% avaient un piercing. Parmi les participantes VIH+, 26 (13,1%) et 22 (11,1%) étaient positives à NG et CT, respectivement, contre 49 (25%) des TS séronégatives au VIH pour chacune de ces deux IST (NG et CT). Une double infection CT/NG a été observée chez 6% des TS séropositives au VIH et chez 14% des séronégatives.

**Tableau 1:** caractéristiques sociodémographiques de 400 travailleuses du sexe à Bamako (Mali), 2020

Caractéristiques	VIH Positif	VIH Négatif	Valeur-p
	N=200; n(%)	N=200; n (%)	
**Âge (années)**			
18-19	12 (6,0)	53 (26,5)	<0,0001
20-24	39 (19,5)	64 (32,0)	
25-29	48 (24,0)	48 (24,0)	
30-34	33 (16,5)	15 (7,5)	
35-39	24 (12,0)	14 (7,0)	
40 et plus	44 (22,0)	6 (3,0)	
**Ancienneté dans le travail du sexe (mois)^†^**			
<12	6 (3,0)	17 (8,5)	0,0013
12-<24	17 (8,5)	33 (16,5)	
24-<48	66 (33,2)	64 (32,0)	
48 et plus	110 (55,3)	86 (43,0)	
**Principal lieu de sollicitation des clients**			
Bar	145 (72,5)	179 (89,5)	<0,0001
Hôtel/Boîte de nuit	28 (14,0)	13 (6,5)	
Autres	27 (13,5)	8 (4,0)	
**Âge au 1^er^ rapport sexuel (année)^†^**			
<10	3 (1,6)	4 (2,1)	0,572
11-13	29 (14,7)	21 (10,9)	
14-15	54 (27,4)	61 (31,8)	
16-17	42 (21,3)	50 (26,0)	
18-19	40 (20,3)	33 (17,2)	
20 et plus	29 (14,7)	23 (12,0)	
**Âge au 1^er^ rapport sexuel payant (année)**			
<18	34 (17,0)	66 (33,0)	1,239
18 et plus	166 (83,0)	134 (67,0)	
**Sources de revenu**			
Travail du sexe uniquement	91 (45,5)	115 (57,5)	0,046
Petit commerce au marché	65 (32,5)	39 (19,5)	
Coiffure	14 (7,0)	31 (15,5)	
Autre occupation	30 (15,0)	15 (7,5)	

**^†^**Différences dues aux données manquantes; %: Pourcentage; N: Nombre; VIH: Virus de l'immunodéficience humaine

**Tableau 2:** caractéristiques comportementales et biologiques de 400 travailleuses du sexe à Bamako (Mali), 2020

Caractéristiques	VIH Positif	VIH Négatif	Valeur-p
	N=200; n(%)	N=200; n(%)	
**Nombre de partenaires sexuels payants au cours des 7 derniers jours^†^**			
0	--	--	<0,0001
1 - 2	44 (22,4)	17 (8,5)	
3 - 4	47 (24,0)	37 (18,5)	
5 - 9	54 (27,6)	51 (25,5)	
10 et plus	51 (26,0)	95 (47,5)	
**Utilisation du condom avec le dernier client^†^**			
Non	1 (0,5)	2 (1,0)	0,380
Oui	199 (99,5)	197 (99,0)	
**Utilisation de drogue injectable^†^**			
Non	198 (99,0)	197 (99,0)	0,652
Oui	2 (1,0)	2 (1,0)	
**Scarification^†^**			
Non	145 (72,5)	161 (80,9)	0,026
Oui	55 (27,5)	38 (19,1)	
**Tatou**			
Non	126 (63,0)	76 (38,2)	<0,0001
Oui	74 (37)	123 (61,8)	
**Piercing**			
Non	173 (86,5)	194 (97,5)	<0,0001
Oui	27 (13,5)	5 (2,5)	
**Vaccin contre l’hépatite B reçu^†^**			
Non	192 (97,0)	181(92,8)	0,413
Oui	6 (3,0)	14 (7,2)	
**Ag HBs^†^**			
Négatif	185 (93,4)	187 (95,4)	0,343
Positif	13 (6,6)	9 (4,6)	
**Ac Anti-VHC^†^**			
Négatif	181 (91,4)	184 (93,9)	0,302
Positif	17 (8,6)	12 (6,1)	

**^†^**Différences dues aux données manquantes; %: Pourcentage; Ac Anti-VHC: Anticorps anti-virus de l’hépatite C; Ag HBs: Antigène de surface de l’hépatite B; N: Nombre; VIH: Virus de l’immunodéficience humaine

**Prévalence de l'Ag HBs et des Ac anti-VHC selon le statut sérologique du VIH:** la proportion des TS positives aux Ag HBs et aux Ac anti-VHC était inférieure à 10%, autant dans le sous-groupe des VIH+ que dans celui des VIH- (Tableau 2).

**Facteurs associés à l'Ag HBs et aux Ac anti-VHC:** l'analyse bivariée a identifié 18 facteurs qui ont atteint le seuil de signification pré-spécifié et qui ont été utilisés pour l'analyse de régression logistique multivarié. Il s'agit de variables relatives aux caractéristiques socio démographiques (âge au premier rapport sexuel payant, statut matrimonial, nombre d'enfants biologiques, religion, source de revenu, ancienneté dans le travail du sexe et principal lieu de sollicitation), comportementales (consommation de boissons alcoolisées, consommation de drogues, nombres de partenaires sexuels payant au cours des sept derniers jours, nombre de partenaires sexuels payant le dernier jour, scarification, tatouage et piercing) et biologiques (NG et CT). L'Ag HBs et l'Ac anti-VHC étaient également associés l'un à l'autre. Dans l'analyse multivariée, six facteurs ont été retenus dont deux avaient une association statistiquement significative avec l'Ag HBs et un avec les Ac anti-VHC (Tableau 3). Concernant l'hépatite B, les participantes qui ont eu le premier rapport sexuel payant avant l'âge de 18 ans étaient plus susceptibles d'avoir un test positif pour l'Ag HBs (RCa; IC95%: 3,3; 1,24-8,63; p=0,017). Les TS ayant un test positif pour les Ac anti-VHC avaient aussi un test positif pour l'Ag HBs: RCa; IC95%: 3,7; 1,21-11,54; p=0,022. Pour l'hépatite C, ce sont les TS positives à l'Ag HBs qui étaient plus fréquemment positives pour l'Ac anti-VHC, comparativement aux TS négatives à l'Ag HBs: RCa; IC95%: 4,1; 1,29-12,56; p=0,016.

**Tableau 3:** facteurs de risque associés à l´antigène HBs et aux anticorps anti-VHC chez 400 travailleuses du sexe à Bamako (Mali), 2020

Caractéristiques	Antigène HBs		Valeur-p	Anticorps anti-VHC		Valeur-p
	n/N (%)	RCa (IC95%)		n/N (%)^†^	RCa (IC95%)	
**Age au 1^er^ rapport sexuel payant**						
<18 ans	9/100 (9,0)	3,3 (1,24-8,63)	0,017	10/100 (1,0)	1,4(0,30-6,41)	0,684
18 ans et plus	13/300 (4,3)	1		19/300 (6,3)	1	
**VIH^†^**						
Positif	13/200 (6,5)	1,5 (0,54-3,96)	0,453	17/200 (8,5)	1,6 (0,71-3,82)	0,251
Négatif	9/199 (4,5)	1		12/199 (6,0)	1	
**Scolarité^†^**						
Non Scolarisée	12/141 (8,5)	2,5 (0,97-6,38)	0,058	10/141 (7,1)	0,3 (0,36-1,88)	0,652
Scolarisée	10/258 (3,9)	1		19/258 (7,4)	1	
**Hépatite B**						
Positif	-	-	-	5/22 (22,7)	4,1 (1,29-12,56)	0,016
Négatif	-	-		24/378 (6,5)	1	
**Hépatite C**						
Positif	5/29 (17,2)	3,7(1,21-11,54)	0,022	-	-	-
Négatif	17/371 (4,6)	1		-	-	
**CT/NG^†^**						
Non	21/292 (7,2)	2,1 (0,62-7,01)	0,235	25/292 (8,6)	0,6 (0,24-1,36)	0,202
Oui	1/107 (0,9)	1		4/107 (3,7)	1	

**^†^**Différences dues aux données manquantes; %: proportion; Anticorps Anti-VHC: Anticorps anti-virus de l'hépatite C; Antigène HBs: Antigène de surface de l'hépatite B; CT: Chlamydia trachomatis; IC95%: Intervalle de confiance 95%; n: numérateur; N: dénominateur; NG: Neisseria Gonorrhoeae; RCa: Rapport de cotes ajusté (ajustement mutuel); VIH: Virus de 'l'immunodéficience humaine

## Discussion

Plus de la moitié des TS séronégatives au VIH (58,5%) avaient moins de 25 ans comparativement au TS séropositives (25,5%). La prévalence du VIH reflétant en bonne partie un risque cumulé à vie, les études montrent que les TS séropositives sont généralement plus âgées que les TS séronégatives au VIH [14,15]. Par ailleurs, dans notre étude, le recrutement sur le terrain qui ciblait les femmes séronégatives à probablement conduit à un sur-échantillonnage de femmes plus jeunes tandis que le recrutement dans les cliniques des femmes séropositives a certainement conduit à une surreprésentation des femmes plus âgées. Les TS séronégatives de notre étude étaient souvent des femmes célibataires, plus éduquées et avec une durée moyenne dans le travail du sexe plus courte, comparativement aux TS séropositives. Par exemple, 55,3% des participantes séropositives au VIH avaient au moins quatre ans d'expérience dans le travail du sexe contre 43% chez les femmes séronégatives. Une étude conduite par Forbi JC *et al*. en 2007 au Nigeria a trouvé des résultats comparables pour le statut matrimonial avec 76,3% de célibataires chez les séronégatives contre 23,7% chez les séropositives [16]. Les TS séropositives au VIH avaient plus d'enfants biologiques dans notre étude puisqu'elles étaient plus âgées (50,5% avaient au moins 30 ans) et avaient souvent vécu maritalement (mariées, divorcées/séparées ou veuves), en comparaison des séronégatives. En moyenne, les TS séronégatives au VIH ont rapporté plus de nouveaux clients et de partenaires sexuels payants au cours des sept derniers jours. Le fait d'être plus jeunes, le plus souvent célibataires, relativement nouvelles dans le milieu du travail du sexe, et de fréquenter plus souvent les bars où on trouve une clientèle diversifiée pourraient expliquer que les TS séronégatives soient davantage sollicitées par les clients. Il est possible aussi que l'infection à VIH se traduise par une relative réduction des activités et du nombre de clients chez certaines TS séropositives [17]. L'utilisation du condom avec le dernier client a été rapportée par la quasi-totalité des TS, indépendamment du statut sérologique vis-à-vis du VIH. Une bonne sensibilisation sur les bénéfices du condom par les agents de santé peut expliquer cela. Des résultats similaires à ceux-ci ont été rapportés chez les TS au Bénin et au Mali [18,19]. En ce qui concerne les IST, différentes études en Afrique de l'Ouest ont montré une fréquence élevée des infections à NG et CT, principalement chez les TS séronégatives au VIH [14,16,19]. Dans notre étude, l'infection à NG a été diagnostiquée chez 13,1% des participantes séropositives au VIH et chez 25% des séronégatives au VIH. Pour l'infection à CT, ces proportions étaient de 11,1% et 25%, respectivement. Les activités de sensibilisation, de dépistage et de traitement des IST aux sein des TS devraient donc se poursuivre. Chez les séronégatives, un intérêt particulier devrait-être porté aux jeunes femmes ayant une expérience limitée dans le milieu du travail du sexe [20].

La prévalence de l'antigène HBs retrouvée chez les TS séropositives au VIH de notre étude était de 6,6%. Chez les donneurs de sang du Mali, la co-infection par le VIH et le VHB est estimée entre 0,1% et 0,5% [21,22]. Au Nigéria, chez des femmes séropositives de la population générale fréquentant l'hôpital national de référence de Jos [2,3], et au Burkina-Faso, chez des femmes enceintes de la population générale [24] et chez des travailleurs de la santé [25], la co-infection VIH-VHB a été estimée à 13%. Cette prévalence est pratiquement le double de celle trouvée dans notre étude. En 2018, à Cotonou, Bénin, Mboup et coll. ont trouvé que 4,5% des femmes TS vivant avec le VIH avaient l'antigène HBs [15]. Cette prévalence correspond à celle trouvée chez les TS séronégatives de notre étude (4,6%). Chez les TS séropositives et séronégatives au VIH, nous avons trouvé une prévalence de 8,6% et 6,1% pour les anticorps anti-VHC, respectivement. Ces prévalences sont élevées par rapport à celles trouvées dans une étude conduite chez des TS au Togo en 2017: 2,6% chez les VIH+ et 5,8% chez les VIH- [26]. Dépendamment de la distribution des facteurs de risque, la prévalence des hépatites virales B et C varie d'une population à l'autre. Par exemple, chez les donneurs de sang, les prévalences du VHB et du VHC étaient respectivement de 18,0% et 9,2% au Burkina Faso en 2012 [27] et de 13,1% et 1,4% au Mali en 2018 [28]. Chez les personnels soignants du Mali en 2018, ces prévalences étaient de 12,1% et 0,6%, respectivement [29]. Les tests de laboratoires qui diffèrent par leur sensibilité et leur spécificité peuvent également contribuer aux écarts observés entre les études [29-32].

Un âge moins avancé au premier rapport sexuel payant (<18 ans; p=0,017) et la présence d'anticorps anti-VHC (p=0,022), étaient les variables associées à la présence de l'antigène HBs. La relation entre l'âge au premier rapport sexuel et les IST en général peut s'expliquer par plusieurs raisons dont une maturité moindre des organes génitaux (la muqueuse de l'exocol est très fragile et sensible aux infections à NG avant la kératinisation consécutive à la première grossesse) et une plus faible capacité des femmes à négocier le condom au cours des rapports sexuels [19]. Seul l'antigène HBs avait une association statistiquement significative avec les anticorps anti-VHC dans le modèle de régression logistique multivarié et ajusté (p=0,017). Les voies communes de transmission des virus du VHB et du VHC expliqueraient ces résultats [28,29]. Au Togo, en 2017, des auteurs ont trouvé une association non significative entre la positivité à l'antigène HBs et les anticorps anti-VHC [25].

Notre étude est l'une des premières à Bamako, au Mali, à estimer la prévalence et les facteurs associés à l'Ag HBs et aux Ac anti-VHC chez les TS. Elle a des limites qui sont à prendre en compte. L'étude transversale ne permet pas de se prononcer objectivement sur le lien de temporalité entre les variables indépendantes et nos deux issues d'intérêt. Cependant, les évidences scientifiques soutiennent les résultats comme l'association entre le début précoce des rapports sexuels et l'antigène HBs [33]. Un sous-rapportage des comportements à risque comme l'utilisation de drogues injectables ou une surestimation de l'utilisation du condom ne sont pas à exclure du fait d'un biais de désirabilité sociale. La discordance entre l'utilisation du condom avec le dernier client qui est extrêmement élevée et la prévalence élevée des IST curables reflète probablement un biais de désirabilité sociale dans l'auto-rapportage de l'utilisation du condom. Pour le diagnostic du VHB, nous avons utilisé comme test de laboratoire le « OnSite HBV-5 Rapid Test » qui recherche cinq marqueurs du VHB (antigène HBs, anticorps anti-HBs, anticorps anti-HBc, antigène HBe, anticorps anti-HBe). Ce test a donné des résultats peu exploitables, sauf pour l'antigène HBs. Ainsi, le fait de ne pas considérer l'infection à vie par le VHB ou l'immunité contre ce virus et de restreindre les analyses à l´antigène HBs seul a nui à la puissance de l´étude et à l´examen en profondeur de l´association entre le VIH et le VHB.

## Conclusion

Les résultats de cette première étude estimant la prévalence et les facteurs associés aux hépatites B et C chez les travailleuses du sexe à Bamako, Mali, indiquent que ces femmes, indépendamment du statut sérologique vis-à-vis du VIH, continuent d´être vulnérables aux infections sexuellement transmissibles en général. Les programmes de contrôle des hépatites B et C chez les TS VIH+ et VIH- doivent se poursuivre et se diversifier dans une perspective de prise en charge intégrée et holistique couvrant la sensibilisation, l´amélioration du dépistage et la vaccination contre le VHB. L´âge de la femme lors des premiers rapports sexuels étant un déterminant important des IST, la prévention et la prise en charge de celles-ci devraient cibler davantage les adolescentes et jeunes filles de la population générale.

### Etat des connaissances sur le sujet

- Les travailleuses du sexe sont vulnérables aux hépatites B et C;
- Les facteurs de risques des hépatites B et C sont multidimensionnels.

### Contribution de notre étude à la connaissance

- La prévalence de l´antigène HBs est plus élevée chez les travailleuses du sexe qui ont eu leur premier rapport sexuel payant avant l´âge de 18 ans;
- L´antigène HBs et les anticorps anti-VHC sont mutuellement associés car partageant des voies communes de transmission;
- Les infections sexuellement transmissibles persistent au sein des TS et il faudrait poursuivre les interventions ciblées pour leur contrôle autant chez les TS VIH+ que chez les VIH-.

## Remerciements

Les auteurs reconnaissent la contribution de toutes les femmes travailleuses du sexe qui ont participé à l'étude et remercient le personnel de ARCAD-SIDA, du centre de recherche et de formation sur le paludisme, et de la cellule sectorielle (du ministère de la Santé) en charge de la lutte contre le Sida (Mali), du dispensaire IST (Centre communal de santé de Cotonou-Zone 1) et de l´Organisation pour la promotion de la santé et le développement communautaire (Bénin), ainsi que du Centre de recherche du CHU de Québec-Université Laval (Canada).
